# P-1679. Discrepancies in antibiotic prescribing: revealing inappropriate use patterns

**DOI:** 10.1093/ofid/ofae631.1845

**Published:** 2025-01-29

**Authors:** Andrea P Silva-Pereira, Anderson F Arias-Ariza, Miguel Ochoa-Vera, Maria L Luna-Gonzalez, Edgar Bernal-Garcia

**Affiliations:** Universidad Autónoma de Bucaramanga, Floridablanca, Santander, Colombia; Universidad Autónoma de Bucaramanga, Floridablanca, Santander, Colombia; Universidad Autónoma de Bucaramanga, Floridablanca, Santander, Colombia; Universidad Autónoma de Bucaramanga, Floridablanca, Santander, Colombia; Universidad Autónoma de Bucaramanga, Floridablanca, Santander, Colombia

## Abstract

**Background:**

Inappropriate antibiotic prescribing fuels antimicrobial resistance (AMR) and compromises patient outcomes. This study aimed to elucidate the prevalence and factors associated with inappropriate antibiotic use across various agents and antibiotic groups.

**Table 1.**
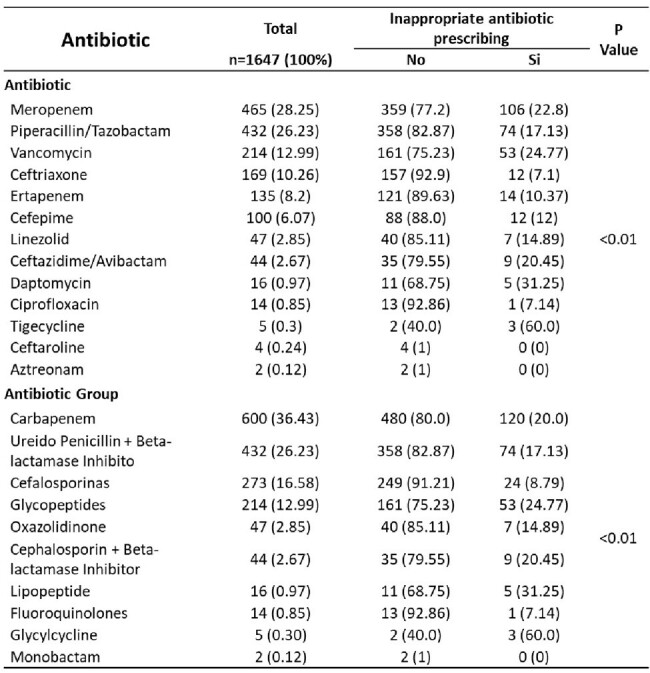
Assessment of Inappropriate Antibiotic Prescribing Patterns by Antibiotic Type

**Methods:**

A retrospective analysis of 1647 cases evaluated antibiotic prescribing patterns. We assessed both prescription rates and prevalence ratios (PR) to examine the appropriateness of antibiotic choice within and across groups.

**Table 2.**
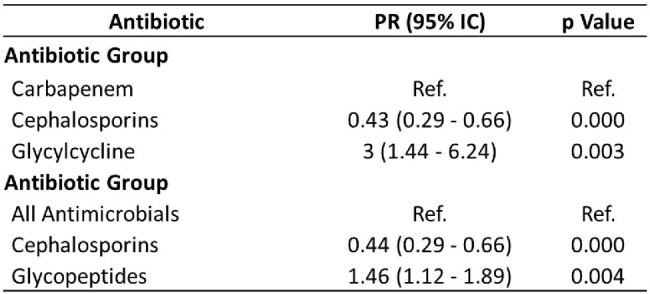
Comparative analysis of prevalence factors for various antimicrobial groups

**Results:**

Prescription rates indicated higher rates of inappropriate use for meropenem (22.8%) and carbapenems (20%) compared to other agents and groups. Conversely, PR analysis revealed a significantly lower likelihood of inappropriate prescribing for cephalosporins compared to carbapenems (adjusted PR: 0.43, 95%CI 0.29-0.66, p < 0.001). Glycylcyclines exhibited a markedly higher risk of inappropriate use (adjusted PR: 3.00, 95%CI 1.44-6.24, p=0.003), while glucopeptides also demonstrated a propensity for inappropriate prescribing (adjusted PR: 1.46, 95%CI: 1.12-1.89, p=0.004).

**Conclusion:**

our study reveals notable discrepancies in the appropriateness of antibiotic prescriptions across different agents and antibiotic groups. While the analysis based on prescription rates demonstrated higher rates of inappropriate use for meropenem and carbapenems overall, prevalence ratio analysis highlighted specific trends within antibiotic classes. Cefalosporins consistently demonstrated a lower likelihood of inappropriate prescribing compared to carbapenems, suggesting their preferential use in clinical practice. However, caution is warranted with glicylcyclines, which exhibited a higher risk of inappropriate prescription. These findings underscore the importance of tailored interventions to optimize antibiotic prescribing practices, mitigate antimicrobial resistance, and improve patient outcomes.

**Disclosures:**

**All Authors**: No reported disclosures

